# Supplementary material for: Assessment of Characteristics of Imaging Biomarkers for Quantifying Anterior Cingulate Cortex Changes: A Twin Study of Middle- to Advanced-Aged Populations in East Asia
Source: Medicina (Kaunas). 2022 Dec 16;58(12):1855. doi: 10.3390/medicina58121855 (PMC9783013; doi:10.3390/medicina58121855)
Supplement: Supplementary file 1 [file medicina-58-01855-s001.zip › medicina-1955039-supplementary.pdf]

Supplemental data

Figure S1a

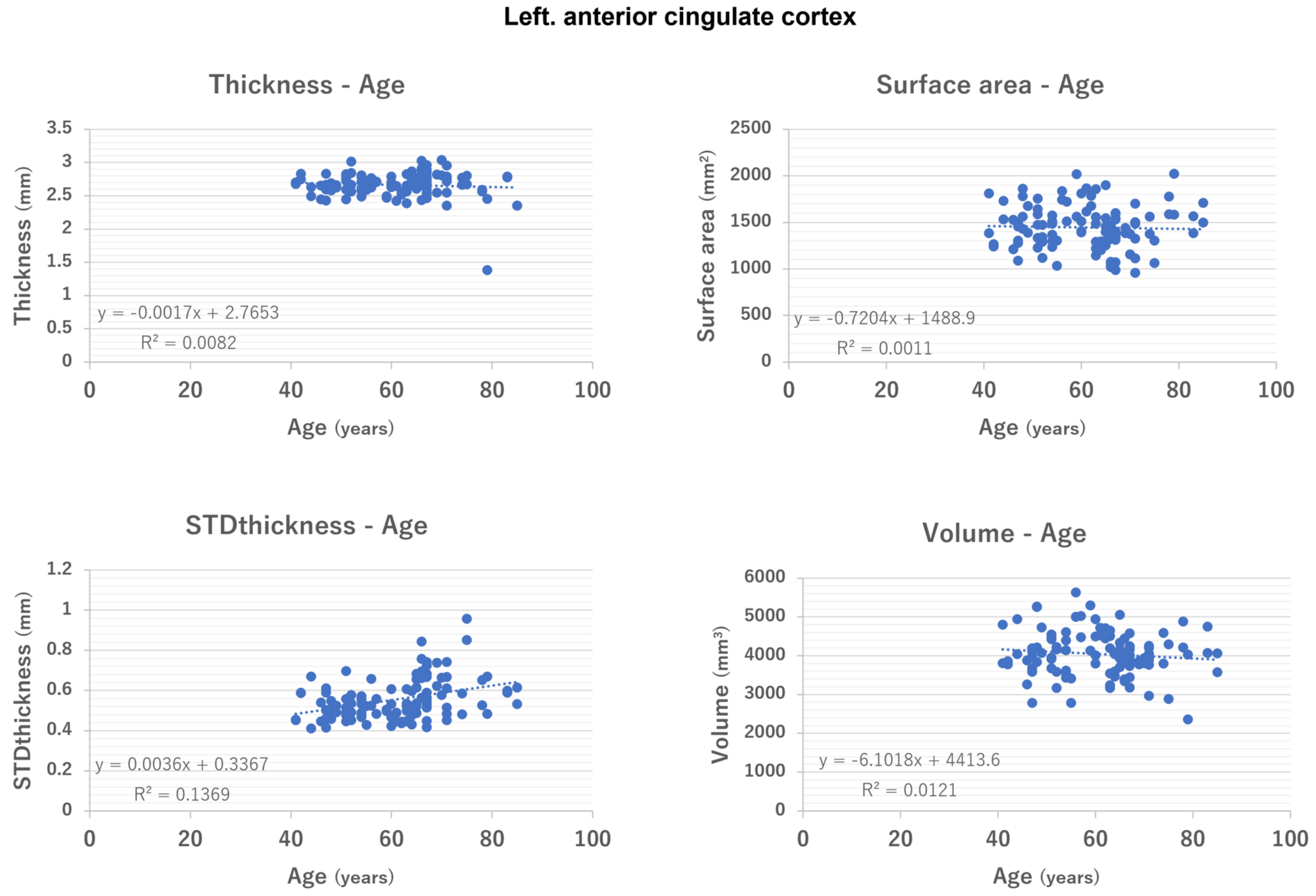
Left. anterior cingulate cortex
Thickness - Age
Thickness (mm)
y = -0.0017x + 2.7653
R² = 0.0082
Age (years)
Surface area - Age
Surface area (mm²)
y = -0.7204x + 1488.9
R² = 0.0011
Age (years)
STDthickness - Age
STDthickness (mm)
y = 0.0036x + 0.3367
R² = 0.1369
Age (years)
Volume - Age
Volume (mm³)
y = -6.1018x + 4413.6
R² = 0.0121
Age (years)

Figure S1b

Left. anterior cingulate cortex

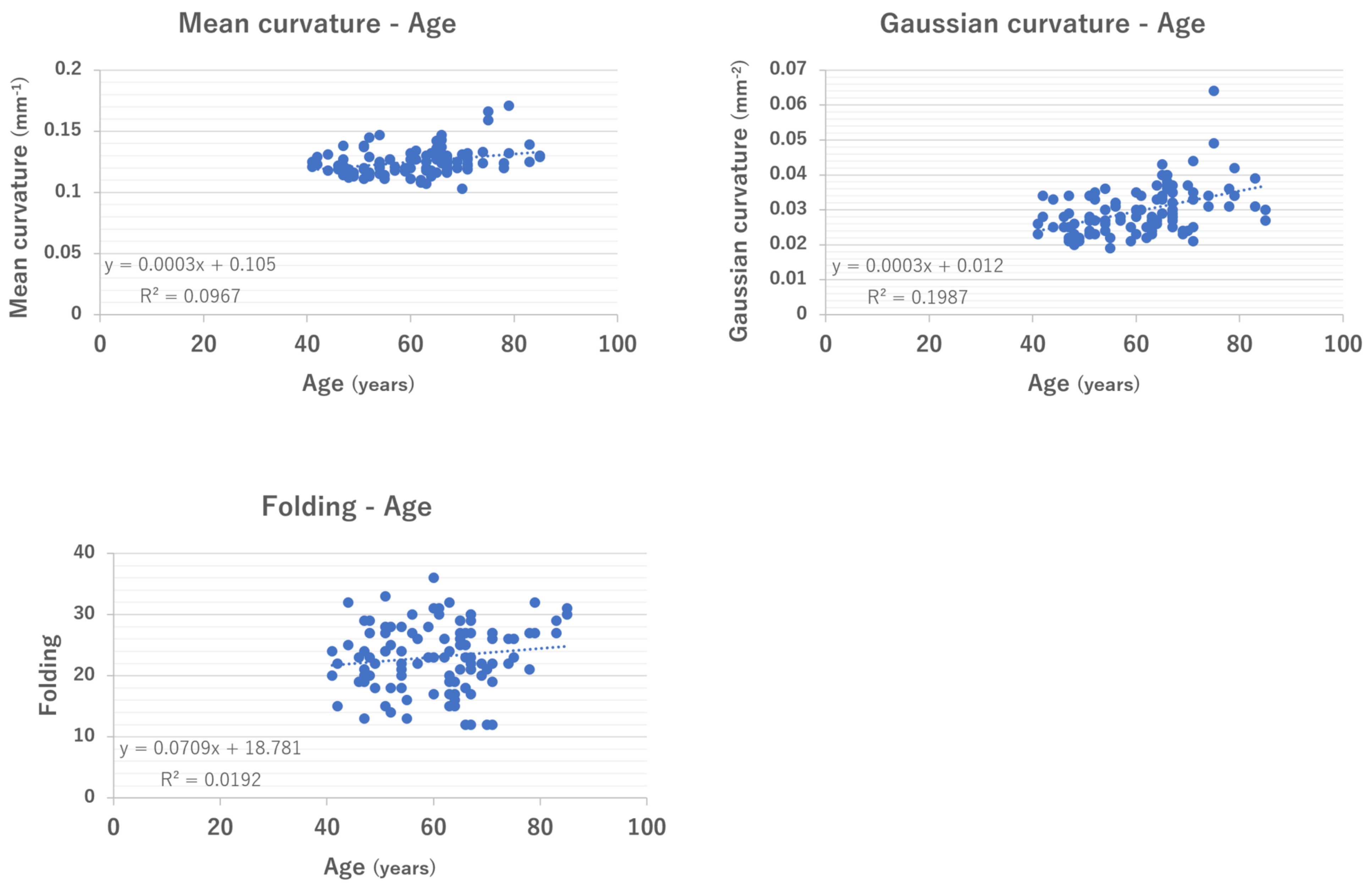

Figure S2a

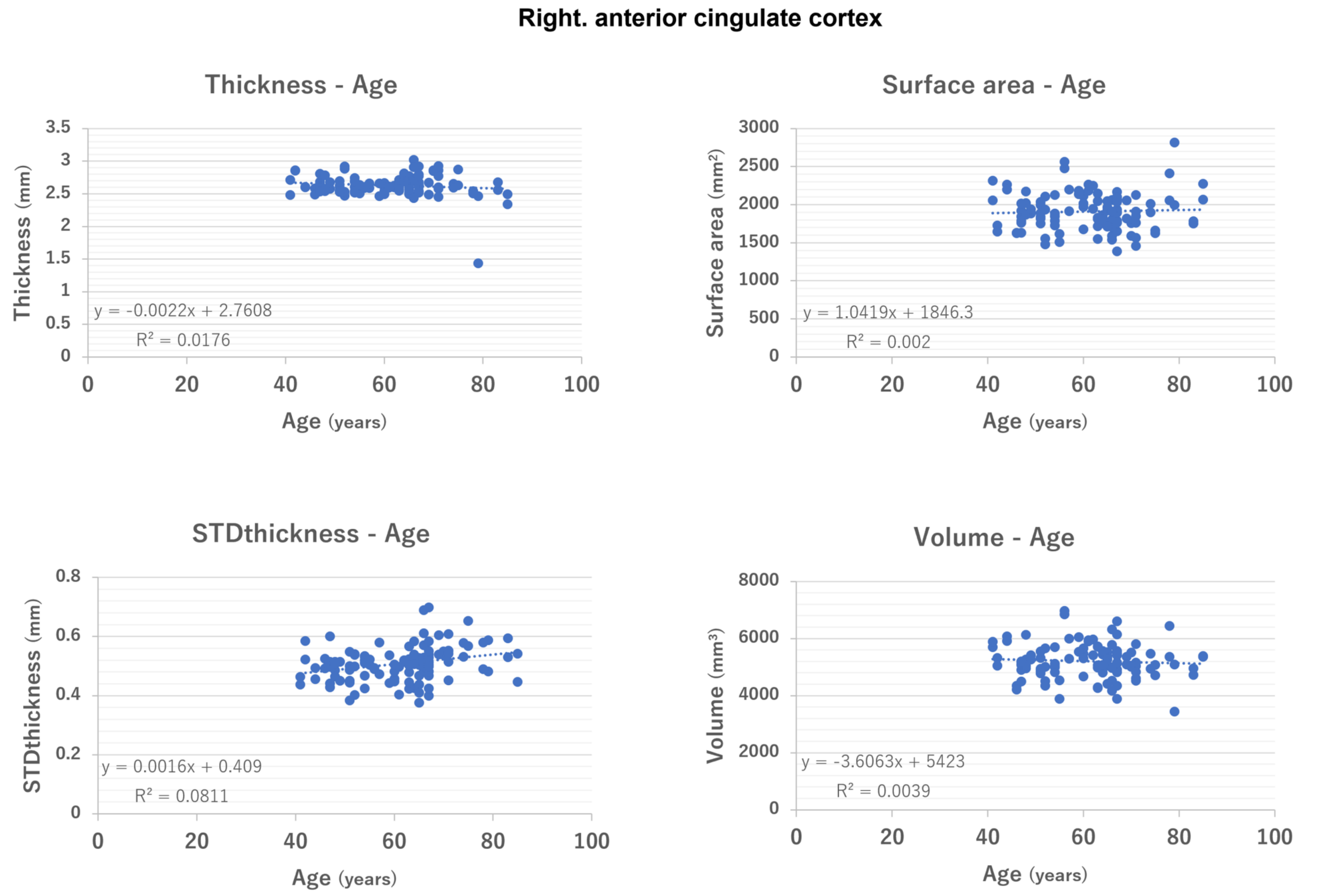
Right. anterior cingulate cortex
Thickness - Age
Thickness (mm)
Age (years)
y = -0.0022x + 2.7608
R^2 = 0.0176
3.5
3
2.5
2
1.5
1
0.5
0
0
20
40
60
80
100
Surface area - Age
Surface area (mm²)
Age (years)
y = 1.0419x + 1846.3
R^2 = 0.002
3000
2500
2000
1500
1000
500
0
STDthickness - Age
STDthickness (mm)
Age (years)
y = 0.0016x + 0.409
R^2 = 0.0811
0.8
0.6
0.4
0.2
Volume - Age
Volume (mm³)
Age (years)
y = -3.6063x + 5423
R^2 = 0.0039
8000
6000
4000
2000

Figure S2b

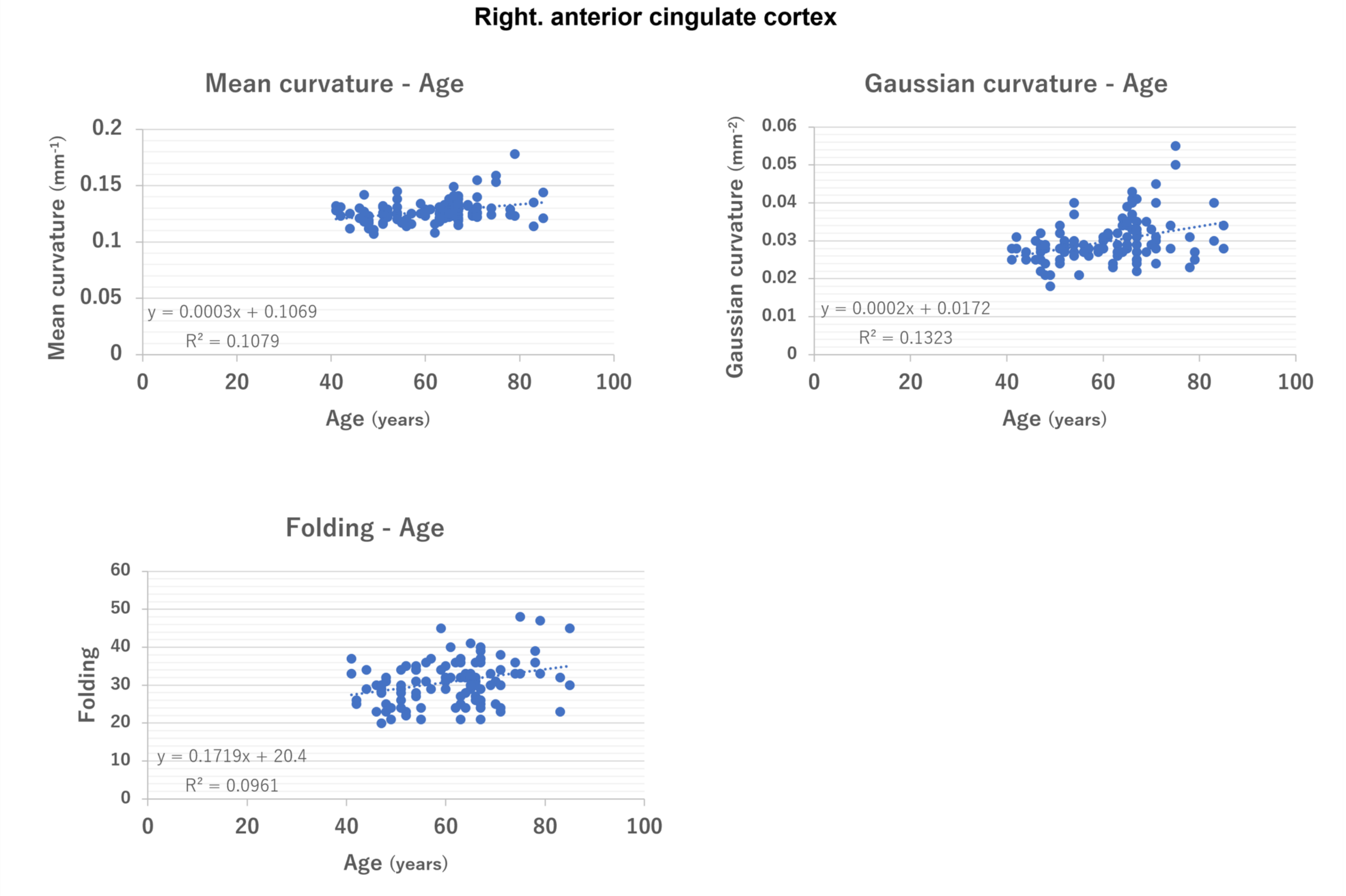

Right. anterior cingulate cortex
Mean curvature - Age
Mean curvature (mm-1)
0.2
0.15
0.1
0.05
0
y = 0.0003x + 0.1069
R² = 0.1079
0
20
40
60
80
100
Age (years)
Gaussian curvature - Age
Gaussian curvature (mm-2)
0.06
0.05
0.04
0.03
0.02
0.01
0
y = 0.0002x + 0.0172
R² = 0.1323
0
20
40
60
80
100
Age (years)
Folding - Age
Folding
60
50
40
30
20
10
0
y = 0.1719x + 20.4
R² = 0.0961
0
20
40
60
80
100
Age (years)

Figure S3a

Left anterior cingulate cortex in Monozygotic twins

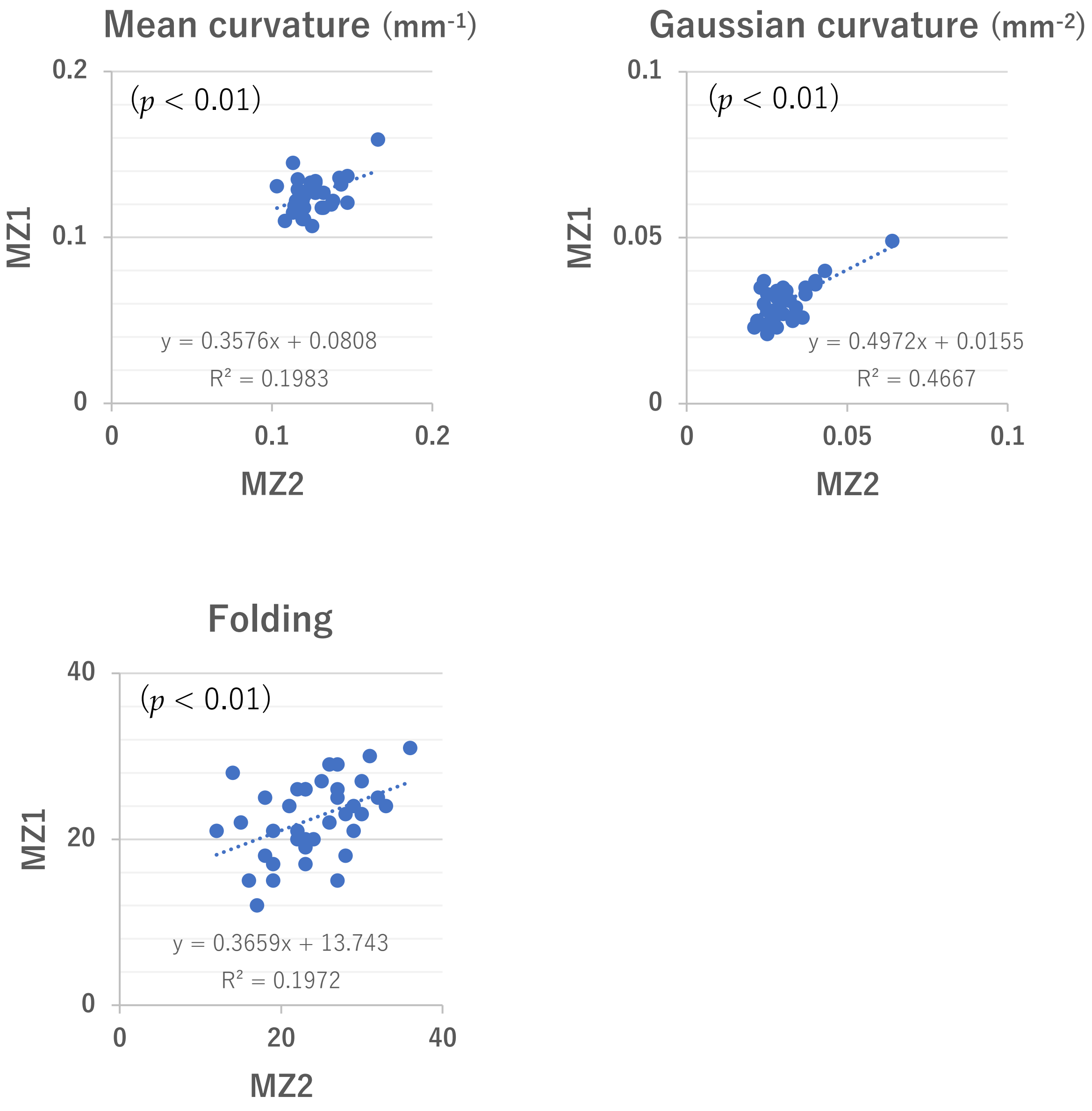

Figure S3b

Left anterior cingulate cortex in Dizygotic twins

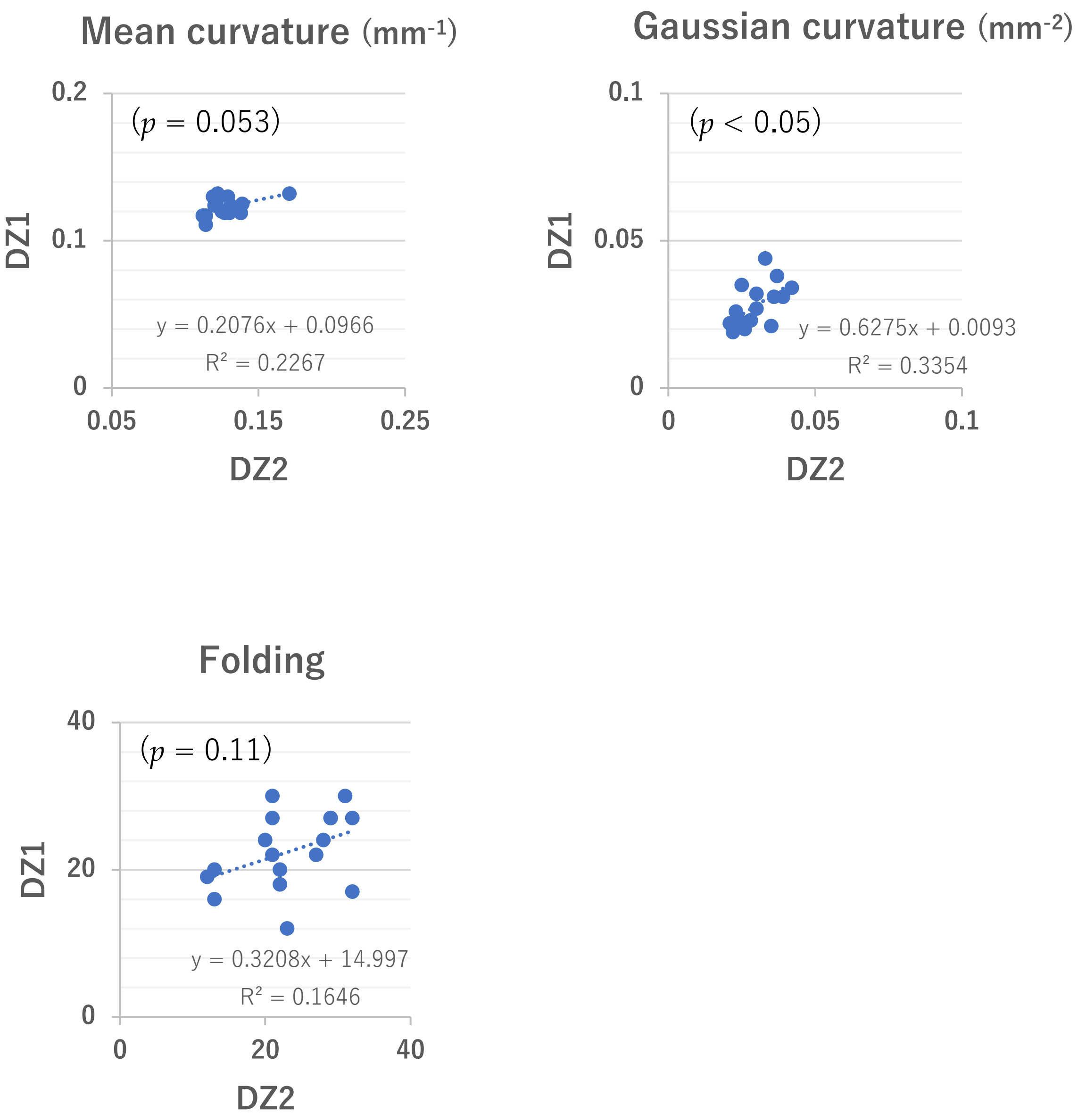

Figure S4a

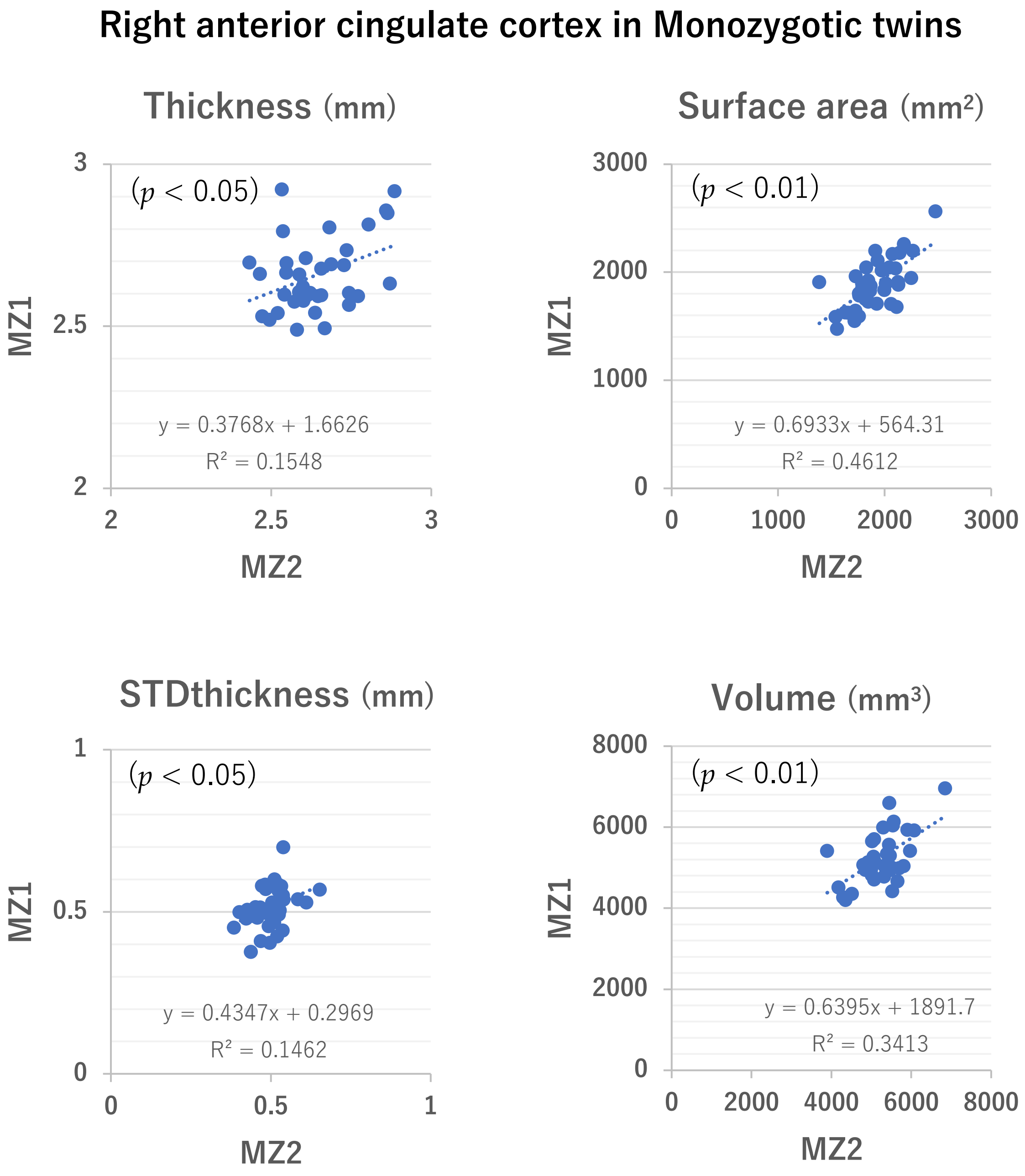
Right anterior cingulate cortex in Monozygotic twins
Thickness (mm)
(p < 0.05)
y = 0.3768x + 1.6626
R² = 0.1548
MZ1
MZ2
3
2.5
2
2
2.5
3
Surface area (mm²)
(p < 0.01)
y = 0.6933x + 564.31
R² = 0.4612
MZ1
MZ2
3000
2000
1000
0
0
1000
2000
3000
STDthickness (mm)
(p < 0.05)
y = 0.4347x + 0.2969
R² = 0.1462
MZ1
MZ2
1
0.5
0
0
0.5
1
Volume (mm³)
(p < 0.01)
y = 0.6395x + 1891.7
R² = 0.3413
MZ1
MZ2
8000
6000
4000
2000
0
0
2000
4000
6000
8000

Figure S4b

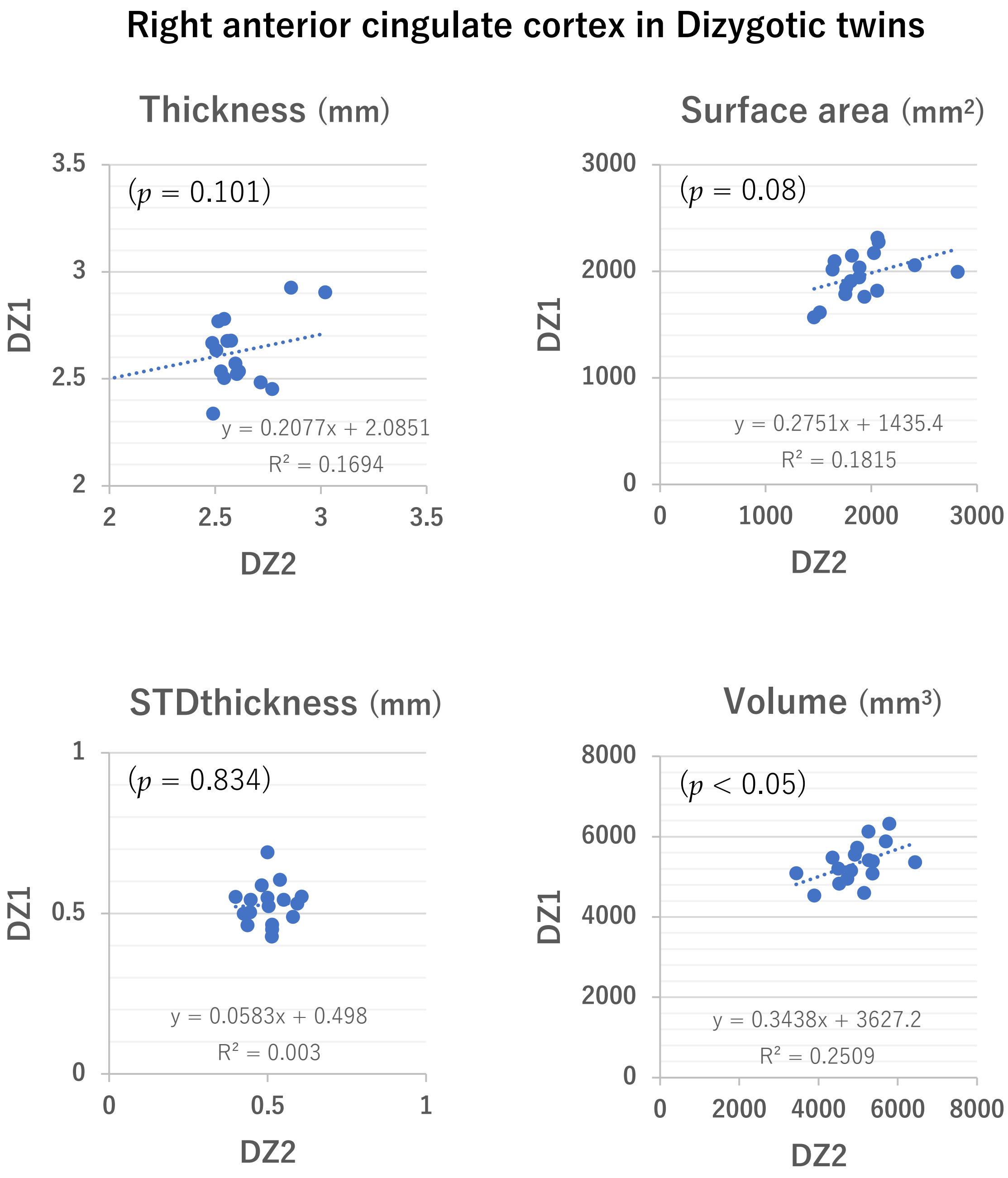

Right anterior cingulate cortex in Dizygotic twins
Thickness (mm)
(p = 0.101)
y = 0.2077x + 2.0851
R² = 0.1694
3.5
3
2.5
2
2
2.5
3
3.5
DZ1
DZ2
Surface area (mm²)
(p = 0.08)
y = 0.2751x + 1435.4
R² = 0.1815
3000
2000
1000
0
0
1000
2000
3000
DZ1
DZ2
STDthickness (mm)
(p = 0.834)
y = 0.0583x + 0.498
R² = 0.003
1
0.5
0
0
0.5
1
DZ1
DZ2
Volume (mm³)
(p < 0.05)
y = 0.3438x + 3627.2
R² = 0.2509
8000
6000
4000
2000
0
0
2000
4000
6000
8000
DZ1
DZ2

Figure S4c

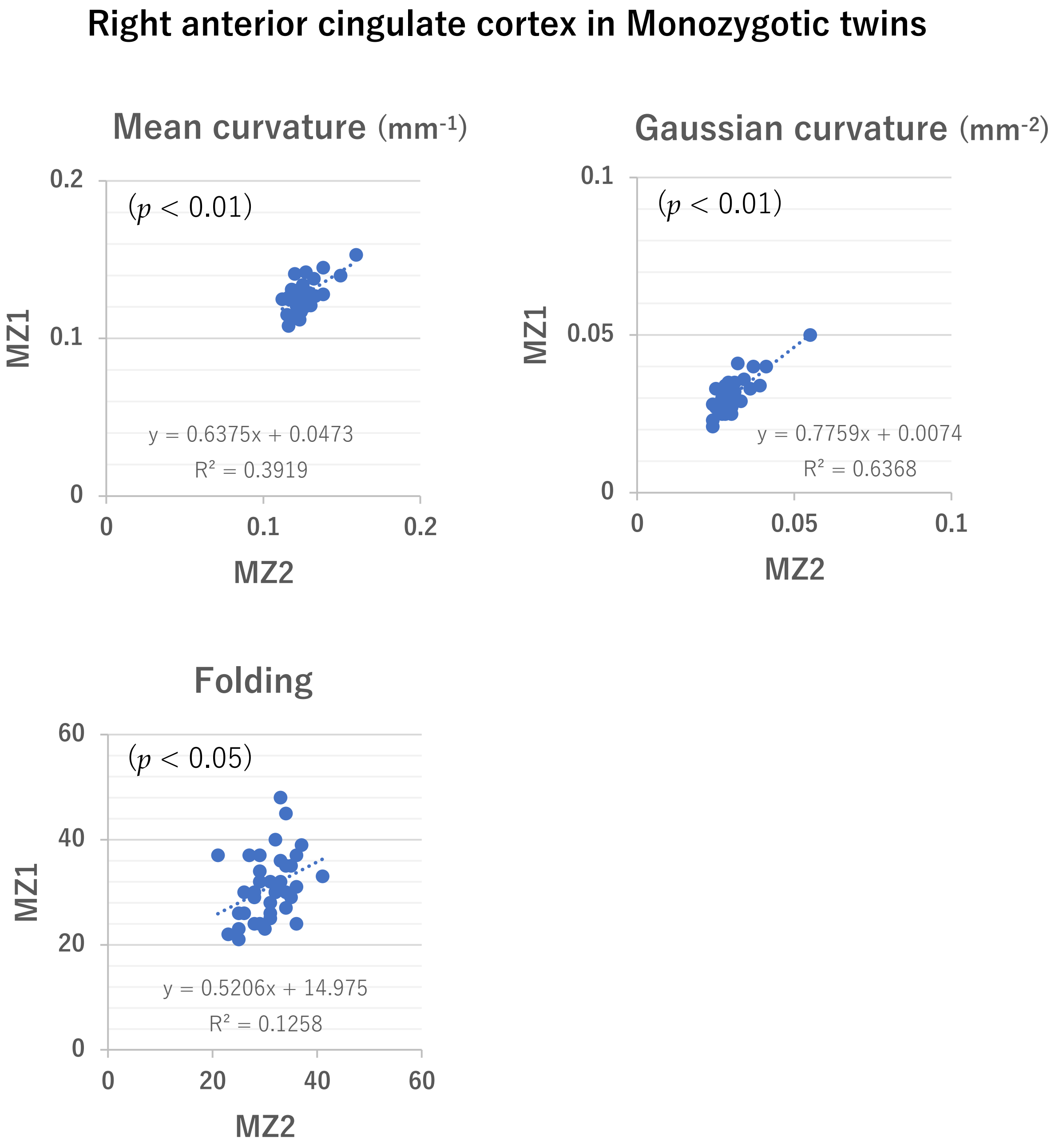
Right anterior cingulate cortex in Monozygotic twins
Mean curvature (mm-1)
(p < 0.01)
y = 0.6375x + 0.0473
R² = 0.3919
MZ1
MZ2
0.2
0.1
0
0
0.1
0.2
Gaussian curvature (mm-2)
(p < 0.01)
y = 0.7759x + 0.0074
R² = 0.6368
MZ1
MZ2
0.1
0.05
0
0
0.05
0.1
Folding
(p < 0.05)
y = 0.5206x + 14.975
R² = 0.1258
MZ1
MZ2
60
40
20
0
0
20
40
60

Figure S4d

Right anterior cingulate cortex in Dizygotic twins

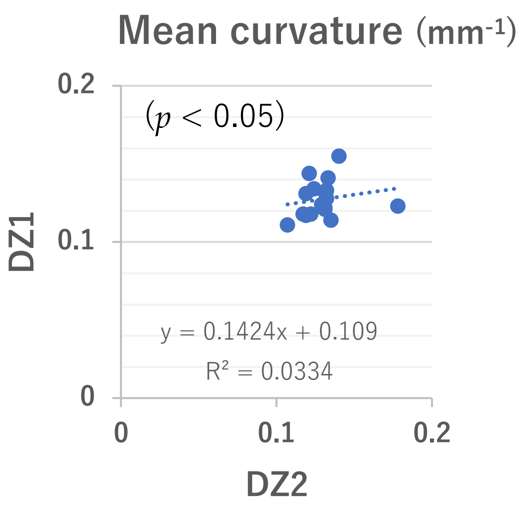

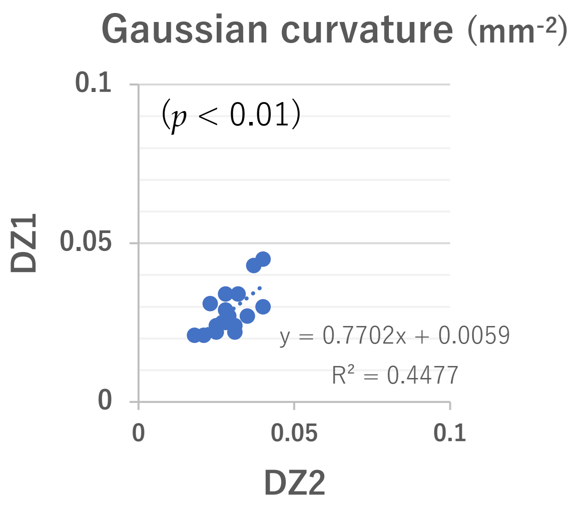

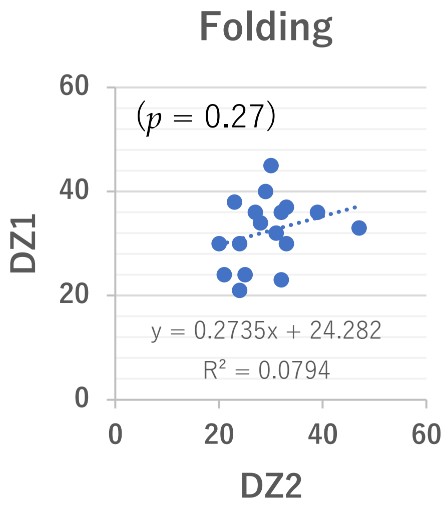

Figure S1 Surface morphological parameter values of the left anterior cingulate cortex correlated with age. (a) Thickness, STDthickness, surface area and volume. (b) Mean curvature, gaussian curvature and folding.

Figure S2 Surface morphological parameter values of the right anterior cingulate cortex correlated with age. (a) Thickness, STDthickness, surface area and volume. (b) Mean curvature, gaussian curvature and folding.

Figure S3 (a) Intra-twin pair correlation of surface morphological parameter values (Mean curvature, gaussian curvature and folding) of the left anterior cingulate cortex in Monozygotic twins. (b) Intra-twin pair correlation of surface morphological parameter values (Mean curvature, gaussian curvature and folding) of the left anterior cingulate cortex in Dizygotic twins.

Figure S4 (a) and (c) Intra-twin pair correlation of surface morphological parameter values of the right anterior cingulate cortex in Monozygotic twins. (b) and (d) Intra-twin pair correlation of surface morphological parameter values of the right anterior cingulate cortex in Dizygotic twins.

NOTE. STDthickness: standard deviation of the thickness
